# Pyrylium- and Pyridinium-Based Ionic Liquids as Friction Modifiers for Greases

**DOI:** 10.1021/acsami.4c01750

**Published:** 2024-03-01

**Authors:** Miguel A. Chacon-Teran, Cinderella Moustafa, Joanne Luu, Ashlie Martini, Michael Findlater

**Affiliations:** † Department of Chemistry and Biochemistry, University of California, Merced, California 95343, United States; ‡ Department of Mechanical Engineering, University of California, Merced, California 95343, United States

**Keywords:** ionic liquids, pyrylium, pyridinium, friction modifier, greases, antiwear additive

## Abstract

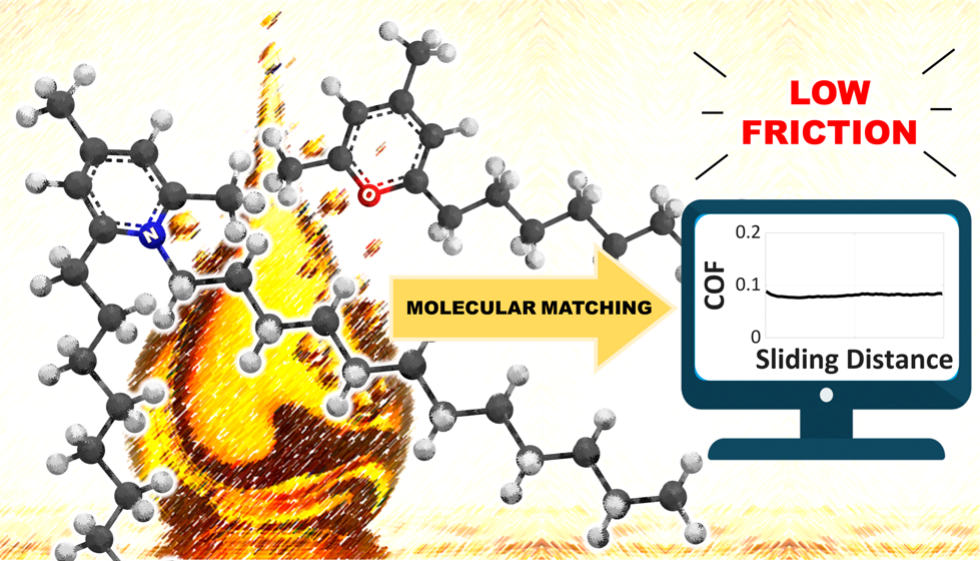

The use of ionic liquids (ILs) as lubricants or additives has been studied extensively over the past few decades. However, the ILs considered for lubricant applications have been part of a limited structural class of phosphonium- or imidazolium-type compounds. Here, new pyrylium- and pyridinium-based ILs bearing long alkyl chains were prepared and evaluated as friction- and wear-reducing additives in naphthenic greases. The physical properties of the synthetic ILs and additized naphthenic grease were measured. The tribological performance of the greases was measured by using standard benchtop tests. The addition of ILs was detrimental to wear, causing an increase in the amount of material removed by sliding relative to the base greases in most cases. In contrast, the friction performance improved under nearly all conditions tested due to the IL additives. The compatibility of the synthetic ILs with the naphthenic greases and its potential influence upon miscibility and tribological performance are tentatively proposed to be a result of the molecular structure.

## 1 Introduction

Minimizing friction energy loss is important for any mechanical system and, as such, is of great interest in the automotive industry due to societal and regulatory pressures geared toward more efficient and “sustainable” modes of transportation.^1^ Reduced friction may directly correspond to higher energy efficiency and increased fuel economy while indirectly contributing to longer-lasting mechanical components.^2,3^ The most straightforward way to minimize friction without changing the components themselves is the use of a low-viscosity lubricant because lower viscosity means less viscous friction. However, as lubricant viscosity is decreased, the interface also moves closer to boundary lubrication, i.e., closer to conditions where the component surfaces are in direct contact, resulting in higher friction and, importantly, loss of material through wear.^2−4^ An attractive approach to solve this problem, which has been explored recently, is the use of ionic liquids (ILs) in lubricant formulations. ILs are low-melting temperature molten salts, typically comprising bulky anions and cations, that have low volatility, are nonflammable, have low melting points, possess high thermal stability, and exhibit a liquid phase over a broad temperature range.^5^ ILs are attractive prospects for lubrication applications because they offer two complementary capabilities: (i) they can be prepared with precisely controlled low viscosity to minimize friction in the full film regime and (ii) their asymmetry enables them to align on component surfaces to provide moderate friction and protection from wear in boundary lubrication.^6^ Due to the inherent polarity of the ILs, most are immiscible in the typically nonpolar hydrocarbon oils. Therefore, most studies have focused on the performance of ILs as additives in nonpolar hydrocarbon oils as oil–IL emulsions,^7,8^ at very low concentrations (<1 wt %),^9,10^ in polar oils like PEG^11−13^ or as base oils.^7,9,14^ There is increasing focus on the design, at a molecular level, of ILs to improve their miscibility with nonpolar oils from both academic and industrial groups.^13,15−23^

**Figure 1:**
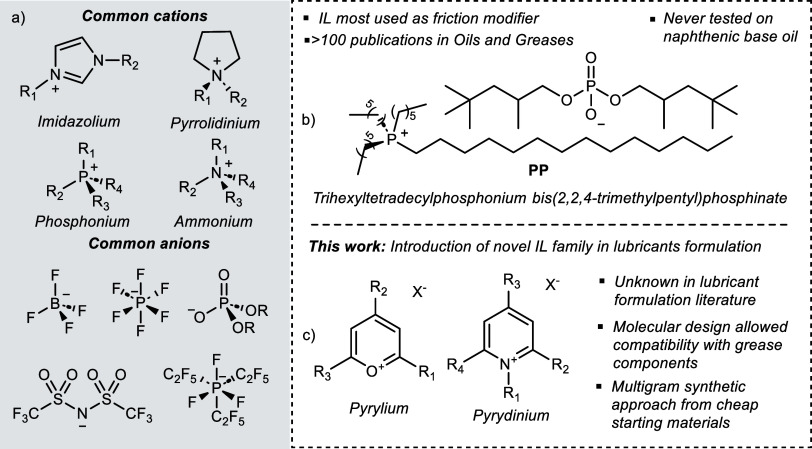
(a) Common cations and anions in oil and grease formulations as friction modifier and antiwear additives. (b) Relevant information related to the IL most studied for its use in lubricant formulations. (c) Summary of the research presented in this work.

Considering the vast array of possible cation/anion pairs, the study and application of ILs as lubricant additives have been quite limited (Figure 1a), especially in greases, likely due to the miscibility problems mentioned above in typical lubricant formulations. Renewed focus on the molecular design of ILs resulted in the development of a family of ILs based upon a quaternary phosphonium cation, composed of long alkyl chains, and a phosphate-based anion partner also bearing (branched) alkyl chains (**PP**, Figure 1b).^15−23^ The number of studies of phosphonium ILs as lubricant additives for oils and greases has increased in recent years, and this class of materials appear to have been established by displaying their best performance as friction modifiers and antiwear additives.^15−23^ Clearly, the molecular design of ILs as lubricant additives is emerging as a promising approach to promote increased compatibility with the components of a formulated lubricating oil or grease.^24^ Such design will enable improvement of the performance of currently known ILs as lubricant additives and provide insights into how it is best to bring novel properties that enhance and/or suppress desirable/undesirable outcomes in newly formulated oils and greases.^16^ In this work, we disclose novel ILs based on pyrylium and pyridinium cations (Figure 1c) and report their performance as friction modifiers or antiwear additives in naphthenic-based greases. To the best of our knowledge, this is the first example of pyrylium and/or pyridinium-based ILs which have been tested for such applications, and we believe that it sets the stage for their expanded use in lubricant formulations.

## 2 Experimental Section

General considerations, characterization data, synthetic experimental details, nuclear magnetic resonance and Fourier transform infrared (FT-IR) spectra, and optical and profilometry wear images are available in the Supporting Information.

### 2.1 Chemicals and Materials

**Figure 2:**
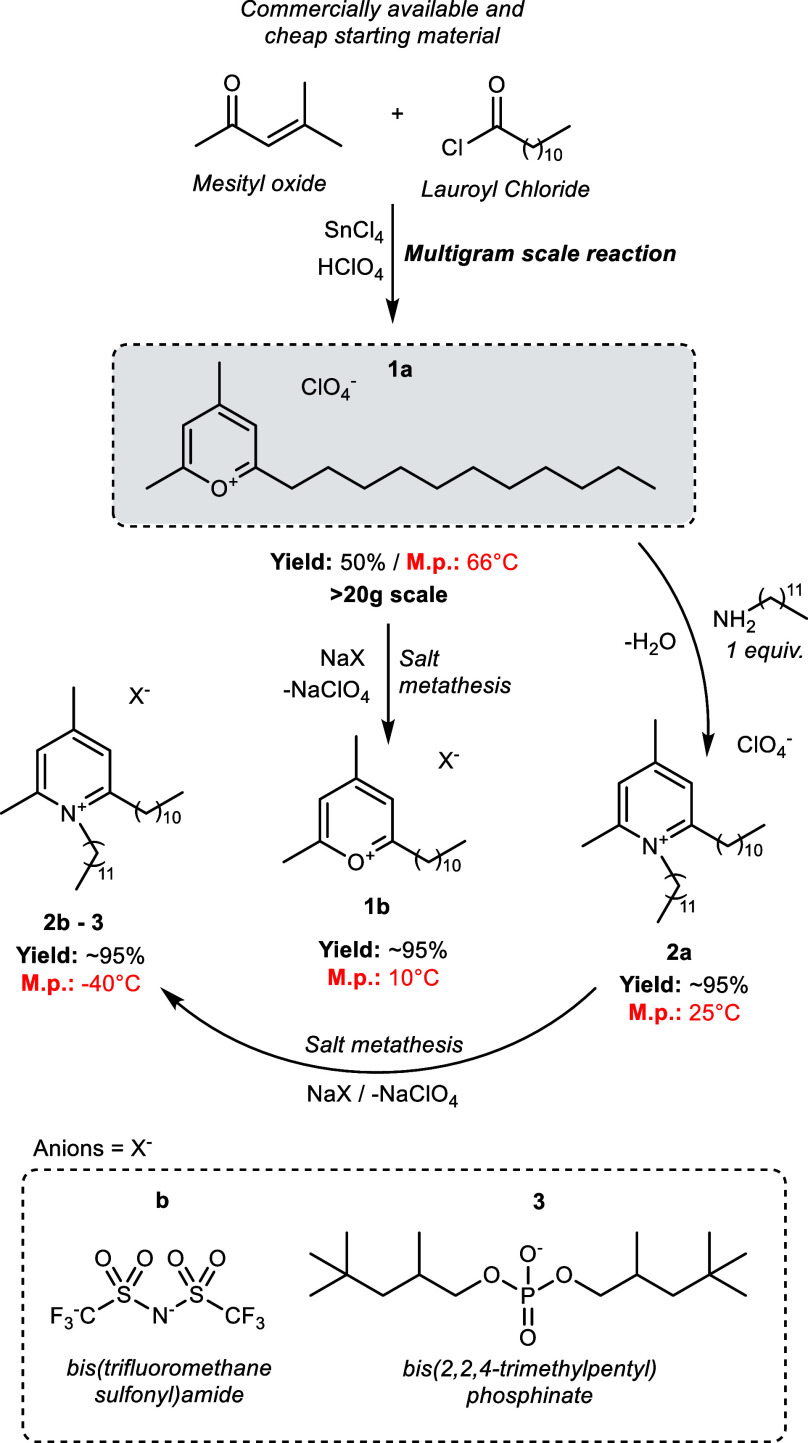
Overall synthetic approach to obtain a novel family of ILs based on pyrylium and pyridinium cores.

The two nonadditized greases used in this work were provided by Nynas AB (Sweden). Table 1 shows the relevant properties of these greases. They were formulated using a naphthenic oil as the base oil and lithium stearate as the thickener (commonly referred to as a lithium complex grease) that was added at 8.55 wt % to form base grease A and at 15.8 wt % to form base grease B. Base grease B also contains poly α-olefins (PAO). ILs **1–3** were synthesized as shown in Figure 2 and used as additives at concentrations of 5 wt % in both base greases. Additionally, greases A and B were blended with 5 wt % of the commercially available trihexyltetradecyl phosphonium bis(2,2,4-trimethylpentyl)phosphinate (**PP**) for comparative purposes. **PP** (>90%) was obtained from Sigma-Aldrich and was degassed prior to use. Chemicals to synthesize ILs such as tin(IV) chloride (98%), lauroyl chloride (98%), mesityl oxide (90%), and dodecyl amine (99%) were obtained from Sigma-Aldrich and degassed prior to use. Additionally, aqueous hydrochloric and perchloric acid were obtained from the same chemical company and were used without further purification. Anhydrous hexane (>95, <0.0001% water content) and dichloromethane (>99.8, <0.0001% water content) were used in synthetic procedures and employed within a dry glovebox MBRAUN workstation. A planetary centrifugal mixer (Intertronics THINKY ARM-310) was used to prepare the grease and the IL blends. The process included two mixing cycles of 5 min at 1600 rpm and a final degassing cycle of 2 min at 2200 rpm.

**Table 1:** Main Properties of the Base Greases Used in This Study

		test grease A	test grease B
base oil		naphthenics	naphthenics + PAO
base oil viscosity, ASTM D445	@ 40 °C, mm^2^/s	561.2	75.1
	@ 100 °C, mm^2^/s	20.6	8.7
thickener (wt %)		lithium stearate (8.55)	lithium stearate (15.8)
NLGI grade		2	2
dropping point, ASTM D2265		>280	>280
oil separation IP121, 40 °C/168 h		3.15	4.63
copper corrosion, ASTM 4048		1A	1B
flow pressure DIN 51805 @ −20 °C, mbar		720	220

### 2.2 Tribological Tests

The IL-additized greases were evaluated by using benchtop friction and wear measurements. Two different types of tests were run: ball-on-disk and four-ball tests.

The ball-on-disk testing was based on standards ASTM D5707-16 and ASTM G99-17. Briefly, the tests involved a 3/8 in. radius 52,100 steel-bearing ball loaded against a rotating 52,100 steel disk, as illustrated in the inset of Figure S18a. The average surface roughness of the ball was 25 nm Ra as purchased. The disk surface was polished to an average roughness of 30–50 nm. The grease was spread uniformly on the disk. Then, a 10 N was applied, corresponding to a maximum Hertz contact pressure of 1 GPa. The rotational speed of the disk was varied depending on the radial position of the ball to achieve a linear sliding speed of 0.25 m/s. The test was run until the total sliding distance reached 400 m. This test was run for each grease at 40 and 100 °C, and select tests were repeated twice. The coefficient of friction (COF) was measured during the tests and wear was measured after the tests using optical microscopy. Figure S18 shows representative results from a ball-on-disk test. The average friction coefficient used to compare different cases was the average of the COF data during the second half of the test. In some cases, there was no visible or measurable wear track on the disk, so wear was calculated as the average diameter of the circular wear patch measured in two orthogonal directions on the ball.

The four-ball tests were run per ASTM-D2266 to characterize wear under extreme pressure (EP) conditions. The 1/2 in. diameter-bearing balls were 52,100 steels with an as-purchased average roughness of 25 nm. One of the balls was loaded against and rotated relative to the other three fixed balls, as illustrated in Figure S19a. As prescribed in the standard, the test was run for 60 min at a rotational speed of 1200 ± 60 rpm and a load of 392 N. The contact pressure per ball was estimated to be 3.45 GPa. The temperature was controlled at 75 °C, and each test was run for 60 min. All four-ball tests were run twice. At the conclusion of the test, the circular worn region on the three fixed balls was measured by using optical microscopy. Representative images of these wear patches are shown in Figure S19b. The average of the circular wear patches measured in two orthogonal directions on all three balls was reported for comparison of the different grease cases.

## 3 Results and Discussion

Our molecular design strategy involved the use of long aliphatic (lipophilic) chains attached to polar (hydrophilic) groups, which contain compounds with tensioactive properties. Such structural design parameters provide an appropriate polarity balance that facilitates interaction with both metal surface and a nonpolar grease. Additionally, the application of novel anions and cations offered the chance to make transformative contributions to the field of ILs. For example, although pyridinium is relatively well known as cationic partners in ILs, the corresponding oxygen-based system, pyrylium (Figure 2, **1a,b**), is scarce and appears in only one study reported in the literature.^25^ Furthermore, applications of either class are unknown in the context of lubricant and grease formulation. Pyrylium and pyridinium cores are both straightforward to prepare synthetically and highly modular, representing an appealing new entry point into IL chemistry.

With these design features in mind, our initial synthetic efforts were focused on the pyrylium compound **1a** (Figure 2) reported by Balaban et al. three decades ago.^26^ The pyrylium salt **1a** was obtained by “SnCl_4_-catalyzed” acylation of mesityl oxide with lauroyl chloride, followed by treatment with perchloric acid. Surprisingly, given the intense interest in the preparation and study of new IL compounds, no subsequent studies have been published. We quickly encountered some challenges in the reported synthetic methodology: (a) the use of stoichiometric quantities of SnCl_4_, (b) a tedious and complex sequence of handling protocols of the reaction mixture, especially the purification step where several recrystallization cycles are required to obtain a pure product, likely arising from the high concentration of SnCl_4_, and (c) a very low overall reaction yield (12% of **1a**). We now report an optimized preparation which delivered improved yield (50% of **1a**) using greatly reduced quantities of SnCl_4_ (20 mol %). Employing these optimized conditions, the isolated pyrylium salt (**1a**) was converted in high yield to the corresponding N-substituted pyridinium salt (**2a**), as shown in Figure 2. In the present work, we also disclose the synthetic diversification of these compounds bearing anions relevant to IL formulation as both friction modifiers and antiwear additives, i.e., bis(trifluoromethanesulfonyl)amide (**1b** and **2b**, Figure 2) and bis(2,2,4-trimethylpentyl) phosphinate (**3**, Figure 2). Moreover, the first use of the inorganic perchlorate anion (**1a** and **2a**, Figure 2) in the context of the IL formulation as lubricant additives is also reported. The derivatization of **1a** and **2a** was accomplished via salt metathesis using one equivalent of the corresponding anion precursor in hexane at room temperature for 6 h. Isolation of the desired compounds was achieved via filtration of the reaction mixture and removal of the reaction solvent under reduced pressure to afford ILs **1b** and **2b** as brownish oils and **3** as a purple oil (detailed procedures describing the synthesis of new ILs and characterization data are available in the Supporting Information).

The newly prepared ILs (**1–3**) exhibited good solubility in nonpolar solvents (e.g., hexane, toluene, etc.), which is a desirable and convenient feature in terms of miscibility between the ILs and the nonpolar base oil greases which are the focus of this work. With novel and commercially available ILs (**1–3** and **PP**, respectively) in hand, the ILs were blended with greases A and B in a planetary centrifuge at a loading of 5 wt %. The newly formulated greases were analyzed by FT-IR and thermogravimetric analysis (TGA)/differential scanning calorimetry (DSC) to explore the chemical compatibility between the ILs and the grease components and the changes in the thermostability of the greases upon addition of the ILs. Infrared spectroscopic analysis showed that the addition of ILs did not directly interact with the functional groups of the components of base greases, inferred by the unaltered IR spectra of blended greases with ILs in comparison with solely base grease (Figures S14 and S15). Subsequently, the thermal behavior of the IL-additized greases was studied on an STA 449 C Jupiter simultaneous TG–DSC instrument from ambient temperature to 600 °C at a heating rate of 10 °C/min in air. The addition of ILs did not interfere with the thermostability features of base greases (Figures S16 and S17). In each case, the addition of ILs resulted in a negligible change in the thermostability of the formulated grease (Figure S17). This is a potentially significant achievement due to the desire to develop additives that enhance a specific physical or chemical feature but do not interfere with previously optimized features such as thermostability.

The performance of the ILs as grease additives was evaluated in terms of their effect on the friction at 40 and 100 °C, wear at 40 and 100 °C, and EP wear at 75 °C. Friction was quantified as the average COF and wear was quantified by the diameter of the circular worn region generated by sliding. The raw data from all tests are reported in the Supporting Information (Figure S20). Here, we evaluated the difference in the performance metrics between base greases A and B (Table 1) and the IL-additized greases. As a reference for these performance metrics, the average base grease EP wear diameter was 0.57 ± 0.02 mm, the average base grease wear diameter was 0.20 ± 0.05 mm, and the average base grease COF was 0.096 ± 0.004 (Figure S20). The performance of the two base greases was similar without ILs. However, grease A exhibited slightly better wear performance at both 40 and 100 °C, while grease B exhibited slightly better friction at both temperatures; these differences are likely attributable to the PAO content in grease B and the corresponding thickener concentrations in the two greases (Figure S20).

Comparing the IL-additized greases to the base greases, on average, the ILs increased wear at both temperatures and in the EP test and decreased friction at both temperatures. This is consistent with previous studies that have shown that ILs are more beneficial for improving friction than wear.^15−23^ This indicates that among the different types of boundary additives in lubricant formulations, these ILs may be more useful as friction modifiers as opposed to antiwear or extreme-pressure additives. As such, we further analyzed only the friction performance of the IL-additized greases, although plots showing the change in wear due to the ILs are shown in Figure S21.

**Figure 3:**
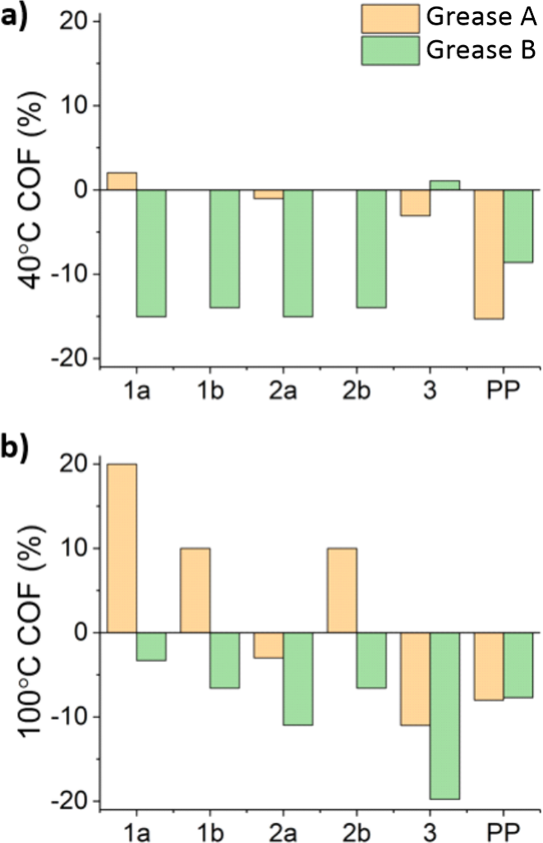
Change in the COF from the ball-on-disk test at (a) 40 and (b) 100 °C resulting from the addition of the various ILs to grease A and grease B. Negative change indicates decreased friction and performance improvement.

Figure 3 shows the change in the COF due to IL addition to grease A and B at 40 and 100 °C. For grease B, all ILs resulted in decreased friction at both 40 and 100 °C, except IL **3** which resulted in slightly increased friction values at 40 °C. For grease A, the ILs had little effect on friction at 40 °C, except IL **PP** which resulted in decreased measured friction values; at 100 °C, only **2a**, **3**, and **PP** were beneficial in terms of friction. The synergy between grease B and the pyrylium and pyridinium ILs may be a result of enhanced compatibility. These results can be rationalized by the presence of PAO in grease B which promotes solvation and/or miscibility of the prepared ILs in the grease mixture. Similar observations have been previously described.^27^ In our study, blending naphthenic grease B with IL 3 resulted in 20% reduction of friction and 10% reduction of wear at 100 °C (Figures 3 and S21). This is a remarkable accomplishment since this is the first report of ILs being used as friction modifiers and/or antiwear additives in naphthenic base greases. We believe that a molecular design approach which focuses on IL structural compatibility can be a potent tool in the expansion of the applicability of ILs as additives in nonpolar base lubricants and greases.

Further analysis revealed that temperature plays a role in the friction performance of the IL-additized greases. For example, there was an improvement in wear performance at higher temperatures (100 °C) in the case of every newly developed IL (Figure S21). However, a similar temperature effect was not observed in friction measurements where **1–2a,b** exhibited poorer performance and only **3** dramatically improved friction performance upon increasing the temperature (from 40 to 100 °C, Figure 3). The behavior shown by **1–2a,b** could be attributed to anion exchange processes between ILs and the thickener (lithium stearate) promoted by the increase in the temperature. Hence, insoluble inorganic salts were formed such as LiClO_4_ for **1–2a** and/or LiN(SO_2_CF_3_)_2_ for **1–2b** as a potential solution. Those inorganic salts are not soluble in nonpolar media, therefore, the formation of salt microparticles in the grease is suspected. Such anion exchange process has been observed before when ILs are formulated with other lubricant additives like zinc dialkyl dithiophosphate.^27^ The formation of inorganic salts has a greater driving force than organic salts, and such a concept is well known in organic synthesis.^28^ Consequently, the presence of salt microparticles would contribute detrimentally to the COF at higher temperatures. It is possible that such microparticles may be acting as a polishing agent, impacting the wear measurements for those cases (ILs **1–2**); however, we do not currently have direct evidence in our system to unequivocally support the presence of polishing.

## 4 Conclusions

For the first time, pyrylium- and pyridinium-based ILs have been explored as friction modifiers and antiwear additives. An optimized, high-yielding, and preparative scale route is reported which will enable the future work with these under-studied ILs. The novel ILs **1–3** were mixed with naphthenic base grease and their friction and wear characteristics were evaluated employing ball-on-disk and four-ball tests. Testing data revealed that these ILs show better performance as friction modifiers rather than as antiwear additives. This is consistent with previous studies that have shown that among the different types of boundary additives in lubricant formulations, ILs tend to be more useful as friction modifiers as opposed to antiwear or EP additives.^15−23^ In particular, the synthetic ILs revealed greater compatibility with grease B, which afforded improved performance at high temperatures. Promisingly, typical grease performance metrics, including thermostability (as determined by TGA), were unaffected by the presence of IL additives, and similar results were obtained, which are typically characteristic of greases which incorporate multiple additives. We speculate that these observations are the result of a better “matching” of the IL molecular structure with that of the greases. This potentially opens new avenues of approach in additive science in which the molecular structures of both IL and grease can be “matched” to provide greater compatibility and performance.

## Supporting Information Available

Synthetic procedures, characterization data of ILs, and tribological measurement details (PDF)
