# Supplementary material for: Pyrylium- and Pyridinium-Based Ionic Liquids as Friction Modifiers for Greases
Source: ACS Appl Mater Interfaces. 2024 Mar 1;16(10):13346–51. doi: 10.1021/acsami.4c01750 (PMC10941177; doi:10.1021/acsami.4c01750)
Supplement: Supplementary file 1 — am4c01750_si_001.pdf [file am4c01750_si_001.pdf]

Supporting information

Pyrylium- and pyridinium-based ionic liquids as friction modifiers for greases.

Miguel A. Chacon-Teran,[a] *Cinderella Moustafa,*[b] *Joanne Luu,* [a] *Ashlie Martini,*[b,*] *Michael Findlater* [a,*]

a. Department of Chemistry and Biochemistry, University of California, Merced, CA 95343. USA. Email: michaelfindlater@ucmerced.edu

b. Department of Mechanical Engineering, University of California, Merced, CA 95343, USA. Email: amartini@ucmerced.edu

KEYWORDS: ionic liquids, pyrylium, pyridinium, friction modifier, greases, anti-wear additive.

Synthetic procedures for ILs

a) Synthesis of **1a**

This synthetic procedure was modified from the original report by Balaban et al. 1992. ***Reaction conditions were optimized to afford higher isolated yield of this compound. NMR data is also provided for the first time.***

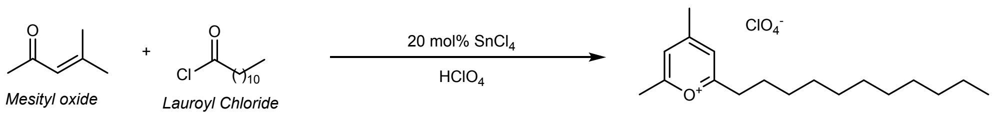

1a

Anhydrous $SnCl_4$ (6.4 g, 24.6 mmol) was added under stirring and with external cooling (ice and water bath) to lauroyl chloride (26.8 g, 123.3 mmol). Then mesityl oxide (10 g, 100.0 mmol) was added so that the temperature was maintained between 22 and 25 °C (the ice was removed from the cooling bath). Stirring was continued after complete addition (seven hours), then the mixture was left to stir overnight. The evolution of hydrogen chloride ceased after final heating for five hours at 40 °C under stirring. The reaction mixture was quenched by pouring over 40 g crushed ice, 4 ml of concentrated aqueous hydrochloric acid and 35 ml diethyl ether. The aqueous layer was separated and extracted twice with 20 ml diethyl ether. The combined organic layers were washed four times with small portions of dilute aqueous hydrogen chloride, after which concentrated aqueous perchloric acid was added. This led to the separation of an upper oily layer which was collected, washed with distilled water, and extracted three times with methylene chloride. These extracts were then dried and concentrated. The resulting oil is transferred to a short, thick, silica gel chromatography column. Excess lauric acid was eluted first flashing the column with hexane after which the pyrylium perchlorate was eluted employing ethyl acetate. From this fraction, after concentration, the pyrylium salt crystallized as colorless micro-crystals (22.1 g), in 50% yield (based on mesityl oxide) having m.p. of 66-67 °C.

^{1}H NMR (500 MHz, Chloroform-*d*) δ 7.76 (s, 1H), 7.65 (s, 1H), 3.13 (t, *J* = 7.9 Hz, 2H), 1.86 (p, *J* = 7.7 Hz, 2H), 1.44-1.28 (m, 18H), 0.88 (t, *J* = 6.9 Hz, 1H).

^{13}C NMR (126 MHz, Chloroform-*d*) δ 181.11, 177.75, 174.51, 124.02, 122.65, 34.94, 31.88, 29.54 (d, *J* = 2.5 Hz), 29.32 (d, *J* = 5.5 Hz), 29.10 (d, *J* = 4.8 Hz), 27.06, 23.95, 22.67, 21.61, 14.11.

b) Synthesis of **1b**

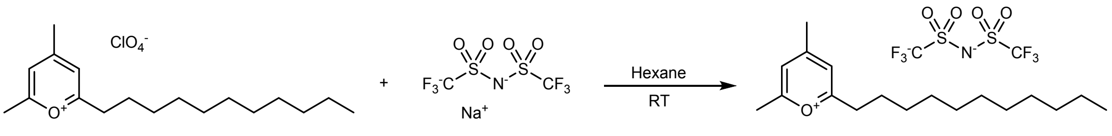

1a (1 equiv.) **1b**

Sodium trifluoromethanesulfonimide (0.30 g, 1.0 mmol) was added portion wise to a hexane solution of **1a** (0.36 g, 1 mmol) at room temperature for 3 h. The resulting suspension was filtered, and solvent was removed under vacuum for 12 h resulting in **1b** as a brownish very viscous oil (0.54 g, 95% yield).

^{1}H NMR (500 MHz, Chloroform-*d*) δ 7.76 (s, 1H), 7.65 (s, 1H), 3.13 (t, *J* = 7.9 Hz, 2H), 1.86 (p, *J* = 7.7 Hz, 2H), 1.44-1.28 (m, 18H), 0.88 (t, *J* = 6.9 Hz, 1H).

^{13}C NMR (126 MHz, Chloroform-*d*) δ 181.11, 177.75, 174.51, 124.02, 122.65, 121.32, 118.13, 114.93, 34.94, 31.88, 29.54 (d, *J* = 2.5 Hz), 29.32 (d, *J* = 5.5 Hz), 29.10 (d, *J* = 4.8 Hz), 27.06, 23.95, 22.67, 21.61, 14.11.

^{19}F NMR (376 MHz, Chloroform-*d*) δ -78.92.

c) Synthesis of **2a**

This synthetic procedure was modified from the original report by Balaban et al. 1992. ***Reaction conditions were optimized to afford higher isolated yield of this compound. NMR data is also provided for the first time.***

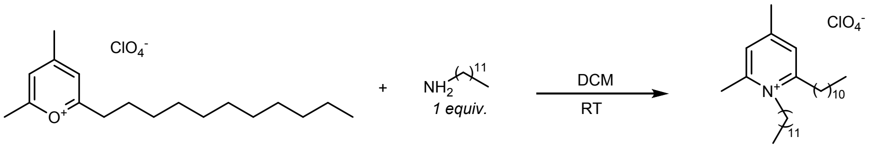

1a **2a**

The pyrylium salt **1a** (1.3 g, 3.6 mmoles) was dissolved in 25 ml dry methylene chloride to which freshly distilled amine (for **5**, 0.78 g, 4.2 mmoles of dodecylamine) were added. The deep red solution was stirred at room temperature for two hours after which acetic acid (0.65 g, 10.8 mmoles) were added. Than an additional 2 hours of stirring is required at 50 °C or until the solution turns yellow. After stirring for another hour, the orange- brown reaction mixture was treated with 50 ml dilute (1%) hydrochloric acid. The methylene chloride layer was washed twice with saturated aqueous ammonium chloride solution (to avoid formation of stable emulsions) and dried over an-hydrous magnesium sulphate. Solvent evaporation left salt **5** (1.7 g, 85% yield) as a waxy oil which crystallized slowly in the refrigerator (m.p. 25 °C).

^{1}H NMR (500 MHz, Chloroform-*d*) δ 7.51 (s, 1H), 7.42 (s, 1H), 4.39 (t, *J* = 7.8 Hz, 2H), 2.96 (t, *J* = 8.4, 7.1 Hz, 2H), 2.83 (s, 3H), 2.53 (s, 3H), 1.77 (m, 4H), 1.46 (m, 4H), 1.34-1.24 (m, 30H), 0.86 (t, *J* = 6.8 Hz, 6H).

^{13}C NMR (126 MHz, Chloroform-*d*) δ 157.66, 157.28, 154.14, 128.93, 127.12, 51.90, 33.07, 31.88, 29.58, 29.46, 29.45, 29.38, 29.32, 29.17, 28.98, 28.67, 26.67, 22.67, 21.69, 21.28.

d) Synthesis **2b**

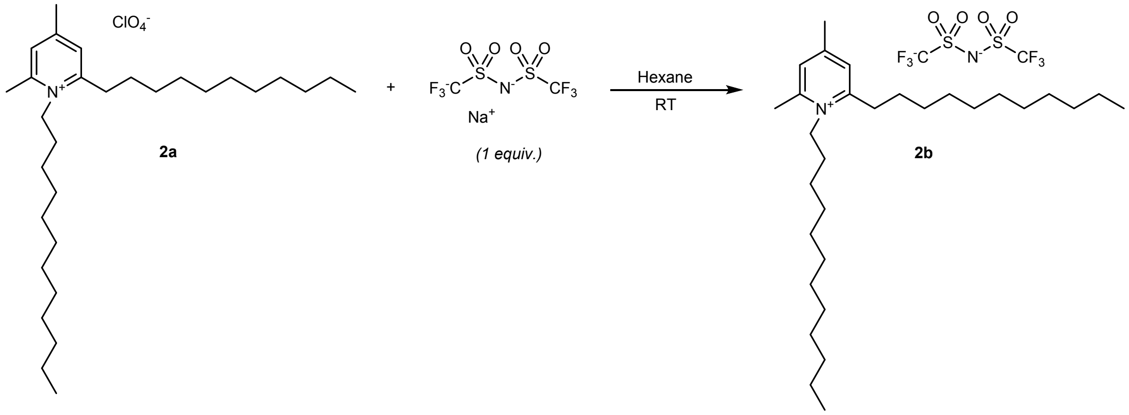

Sodium trifluoromethanesulfonimide (0.30 g, 1.0 mmol) was added portion wise to a hexane solution of **2a** (0.53 g, 1 mmol) at room temperature for 3 h. The resulting suspension was filtered, and solvent was removed under vacuum for 12 h resulting in **2b** as a brownish very viscous oil (0.70 g, 95% yield).

^{1}H NMR (500 MHz, Chloroform-*d*) δ 7.51 (s, 1H), 7.42 (s, 1H), 4.39 (t, *J* = 7.8 Hz, 2H), 2.96 (t, *J* = 8.4, 7.1 Hz, 2H), 2.83 (s, 3H), 2.53 (s, 3H), 1.77 (m, 4H), 1.46 (m, 4H), 1.34-1.24 (m, 30H), 0.86 (t, *J* = 6.8 Hz, 6H).

^{13}C NMR (126 MHz, Chloroform-*d*) δ 157.66, 157.28, 154.14, 128.93, 127.12, 121.37, 118.17, 51.90, 33.07, 31.88, 29.58, 29.46, 29.45, 29.38, 29.32, 29.17, 28.98, 28.67, 26.67, 22.67, 21.69, 21.28.

^{19}F NMR (376 MHz, Chloroform-*d*) δ -78.82.

e) Synthesis **3**

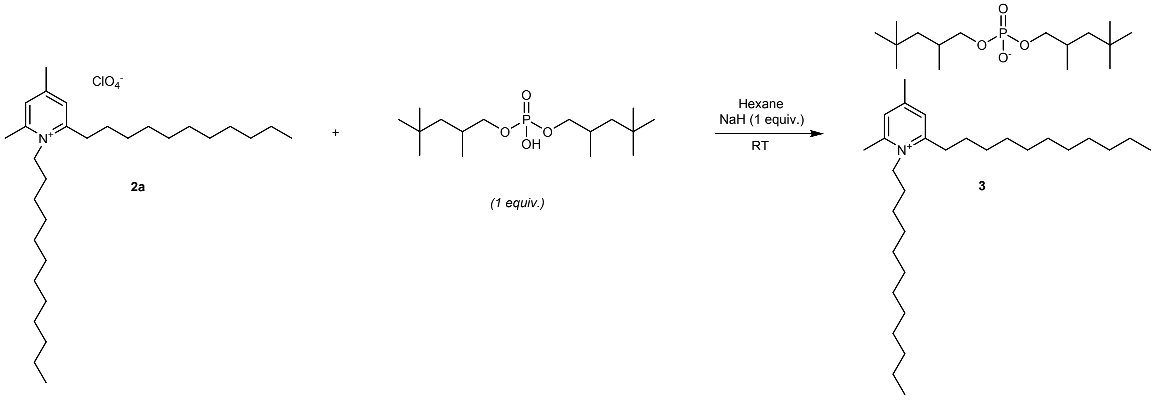

Sodium hydride (0.045 g, 1.89 mmol) was added portion wise to a hexane solution of Bis(2-ethylhexyl) phosphate (0.608 g, 1.89 mmol) at room temperature for 1 h. Then a

solution of **2a** (1 g, 1.89 mmol) in hexane was added dropwise. The resulting suspension was filtered, and solvent was removed under vacuum for 12 h resulting in **3** as a deep purple and very viscous oil (1.35 g, 95% yield).

^{1}H NMR (500 MHz, Chloroform-*d*) δ 7.51 (s, 1H), 7.42 (s, 1H), 4.39 (t, *J* = 7.8 Hz, 2H), 3.94 (m, 4H) 2.98 (t, *J* = 7.1 Hz, 2H), 2.83 (s, 3H), 2.53 (s, 3H), 2.17 (s, 6H), 1.80 (m, 4H), 1.57-1.26 (m, 48H), 0.86 (t, *J* = 6.8 Hz, 16H).

^{13}C NMR (126 MHz, Chloroform-*d*) δ 206.99, 157.60, 157.33, 154.20, 128.91, 127.07, 69.63, 69.58, 51.95, 40.02, 39.96, 33.10, 31.89, 30.91, 29.82, 29.58, 29.48, 29.46, 29.45, 29.39, 29.32, 29.31, 29.17, 29.00, 28.84, 28.64, 26.68, 23.17, 22.94, 22.67, 21.71, 21.31, 14.10, 14.01, 10.85.

^{31}P NMR (202 MHz, Chloroform-*d*) δ 1.78.

NMR spectra for ILs

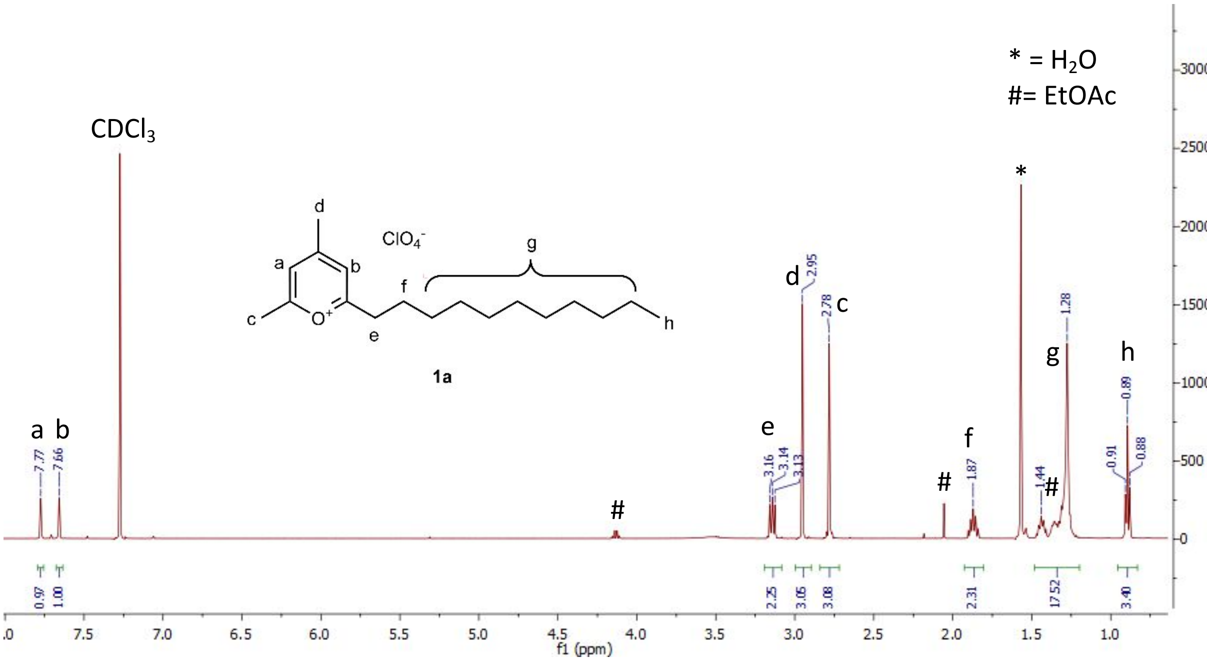

Figure S1. ^{1}H NMR spectrum (500 MHz, $CDCl_3$) of **1a**.

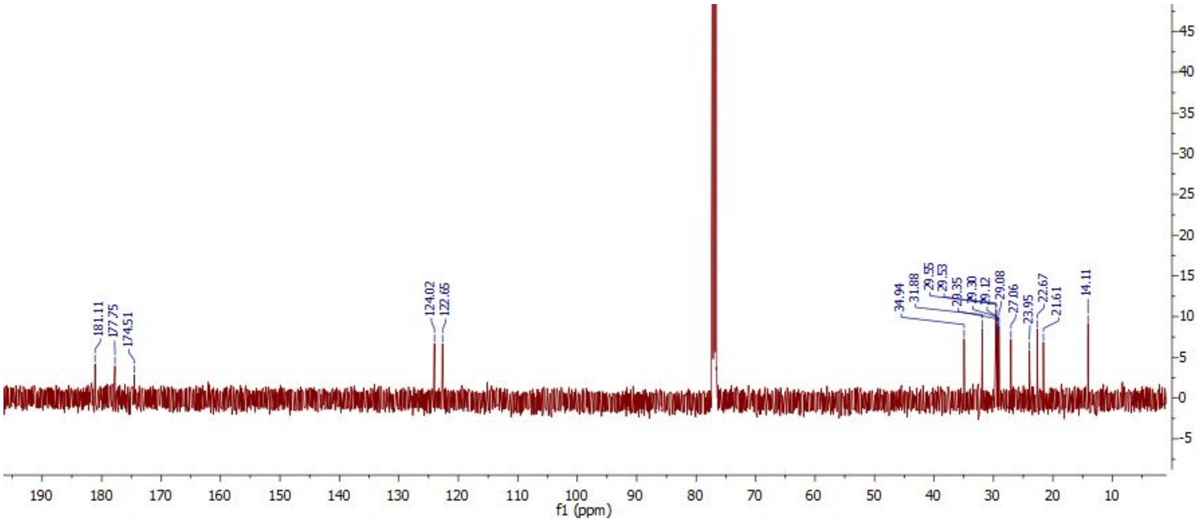
Figure S2. $^{13}C\{^1H\}$ NMR spectrum (126 MHz, $CDCl_3$) of **1a**.

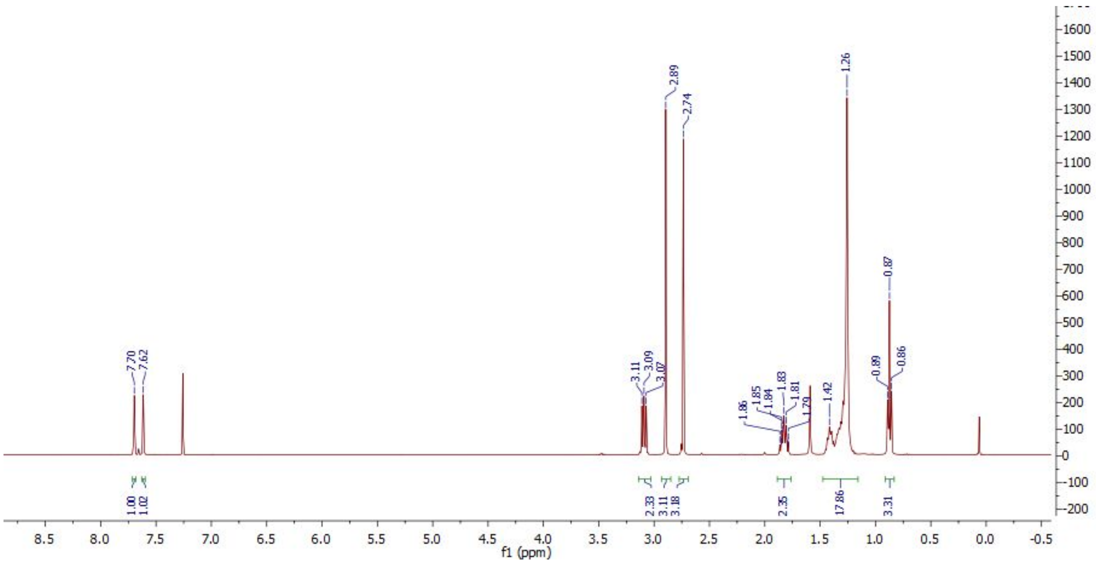

Figure S3. ^{1}H NMR spectrum (500 MHz, $CDCl_3$) of **1b**.

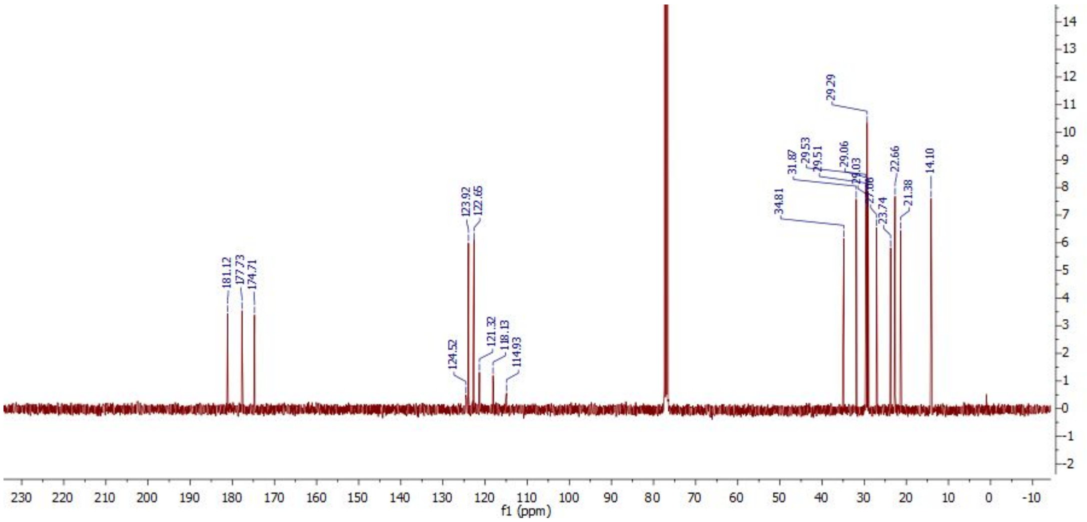

Figure S4. ^{13}C{^{1}H} NMR spectrum (126 MHz, $CDCl_3$) of **1b**.

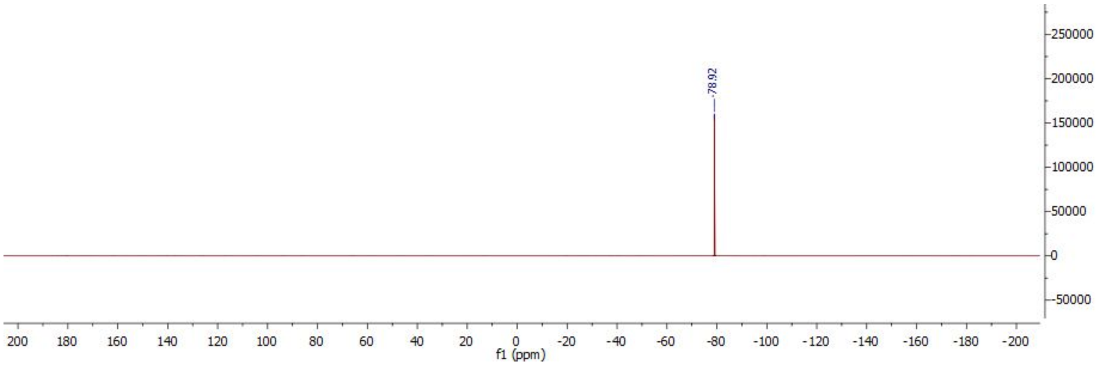

Figure S5. ^{19}F NMR spectrum (376 MHz, $CDCl_3$) of **1b**.

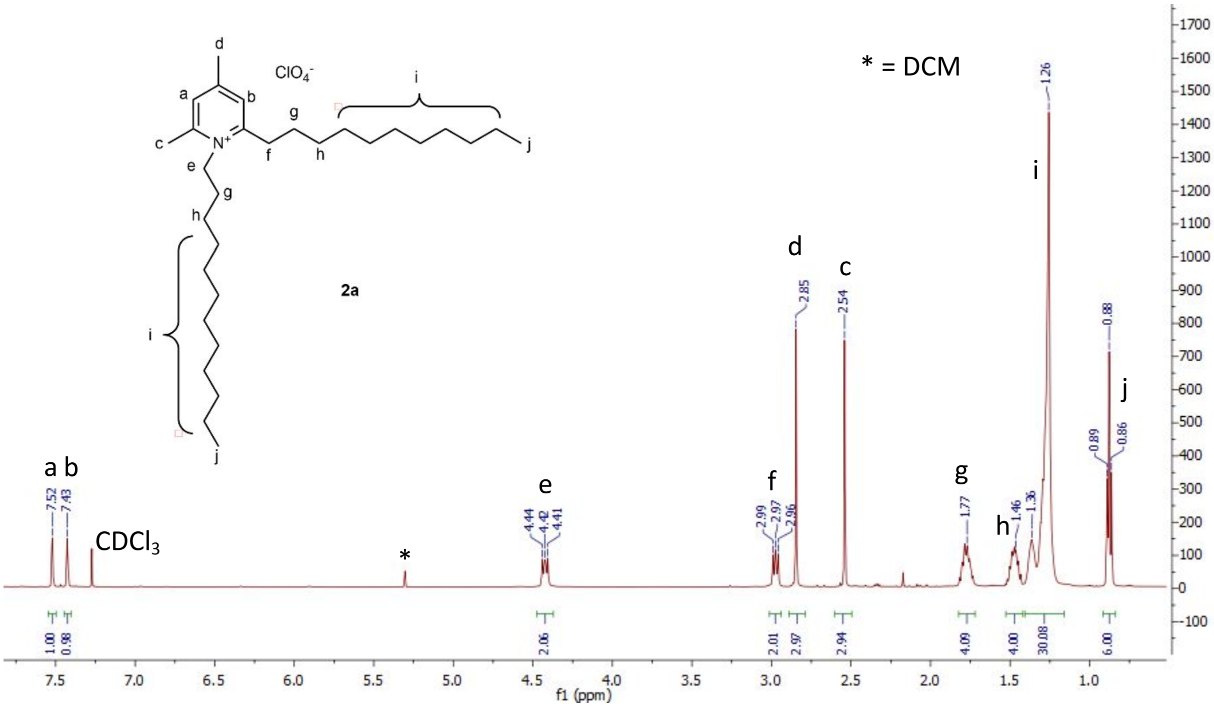

Figure S6. ^{1}H NMR spectrum (500 MHz, $CDCl_3$) of **2a**.

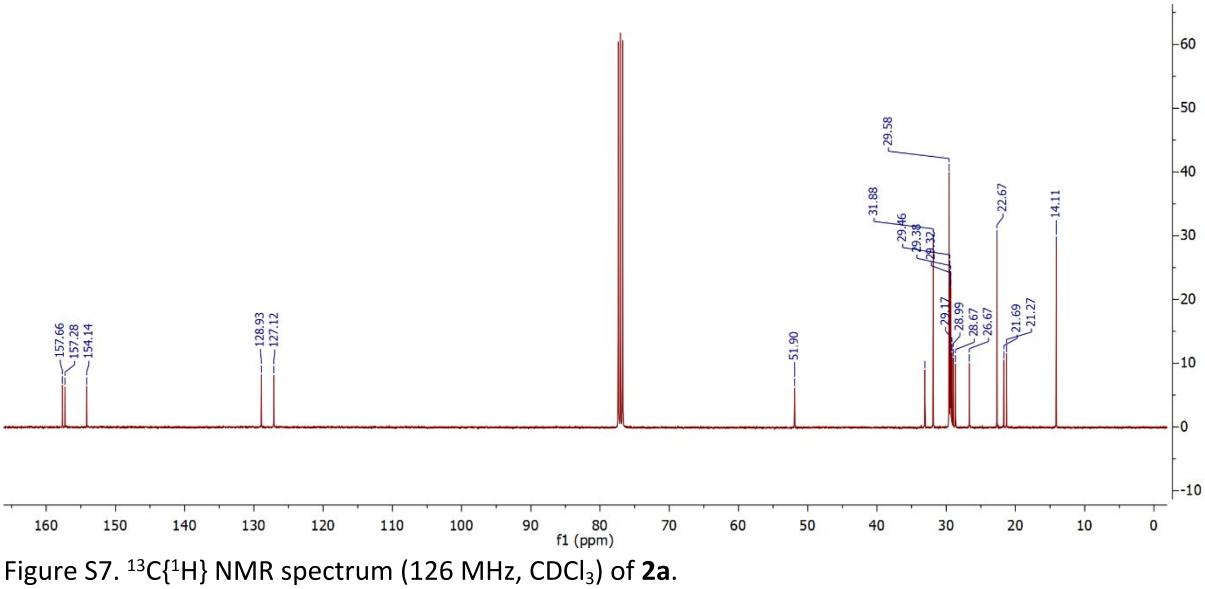

Figure S7. $^{13}C\{^{1}H\}$ NMR spectrum (126 MHz, $CDCl_3$) of **2a**.

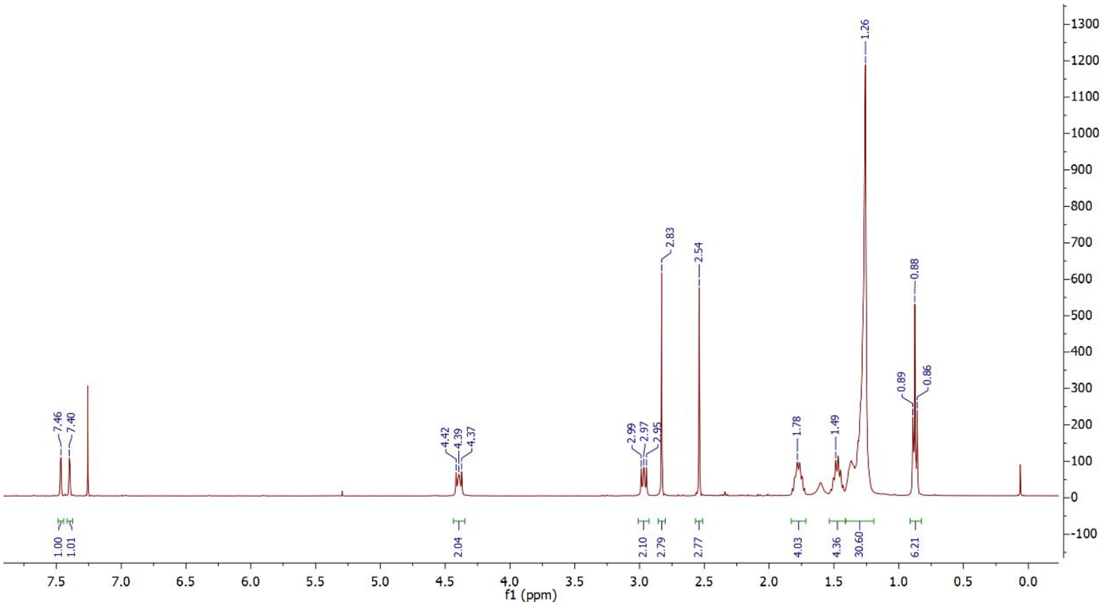

Figure S8. ^{1}H NMR spectrum (500 MHz, $CDCl_3$) of **2b**.

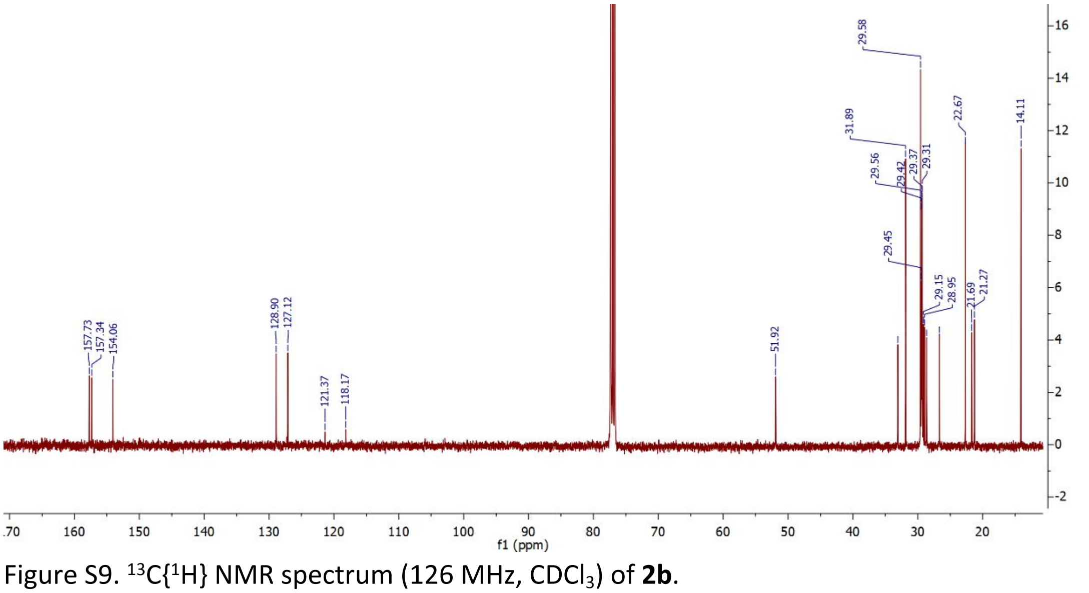

Figure S9. $^{13}C\{^{1}H\}$ NMR spectrum (126 MHz, $CDCl_3$) of **2b**.

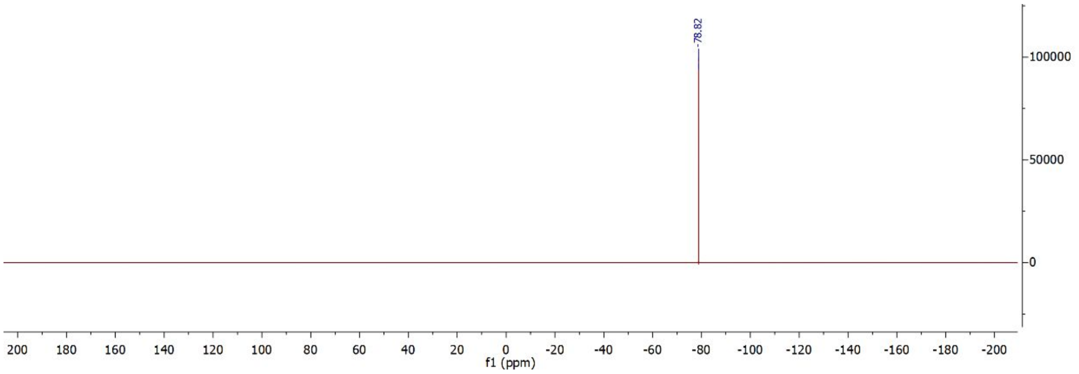

Figure S10. ^{19}F NMR spectrum (376 MHz, $CDCl_3$) of **2b**.

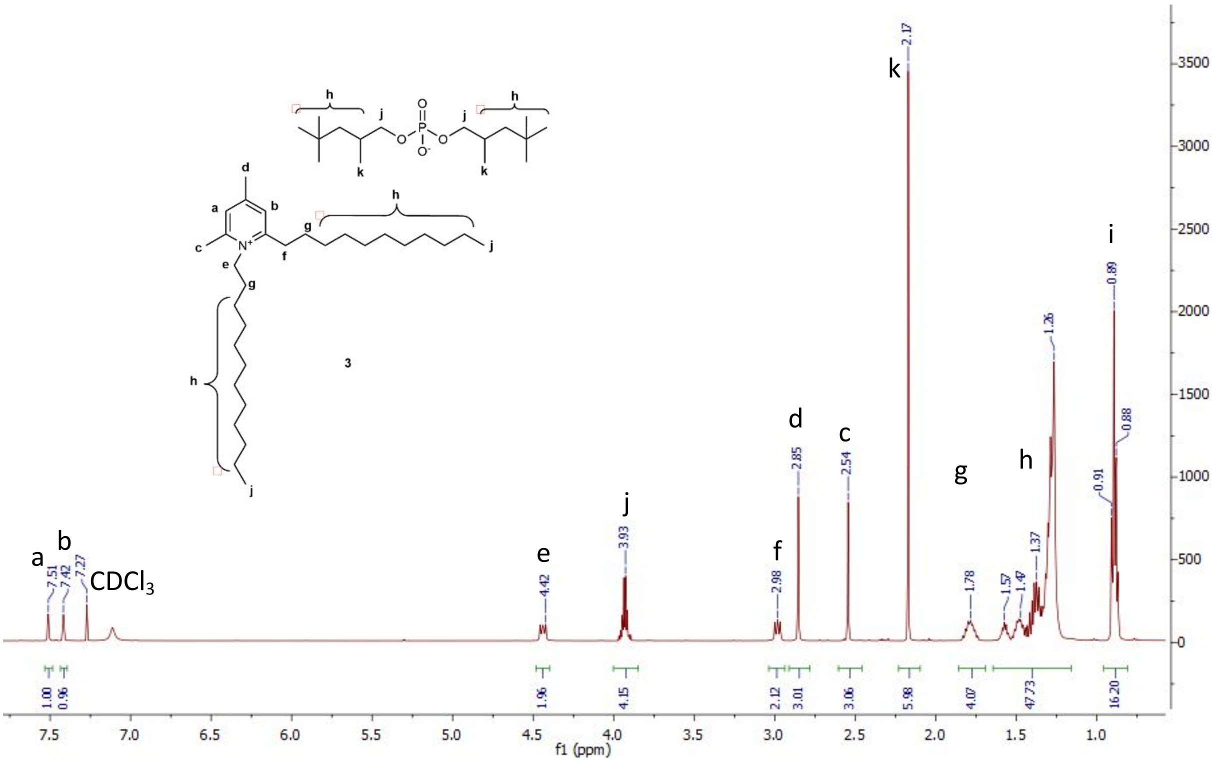

Figure S11. ^{1}H NMR spectrum (500 MHz, $CDCl_3$) of **3**.

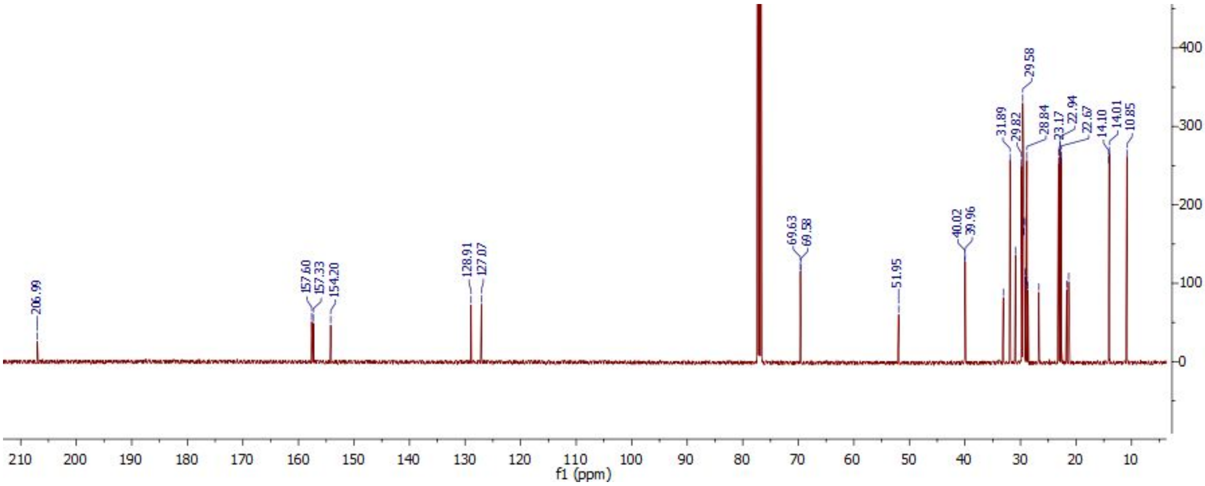

Figure S12. $^{13}C\{^{1}H\}$ NMR spectrum (126 MHz, $CDCl_3$) of **3**.

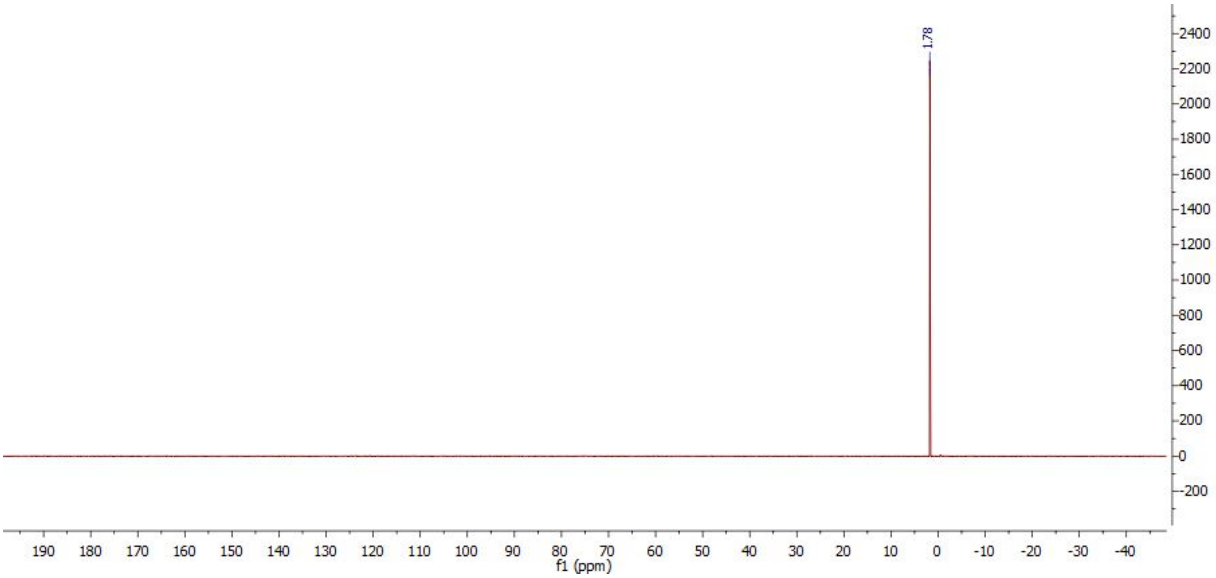

Figure S13. ^{31}P NMR spectrum (202 MHz, $CDCl_3$) of **3**.

FT-IR Spectra of ILs

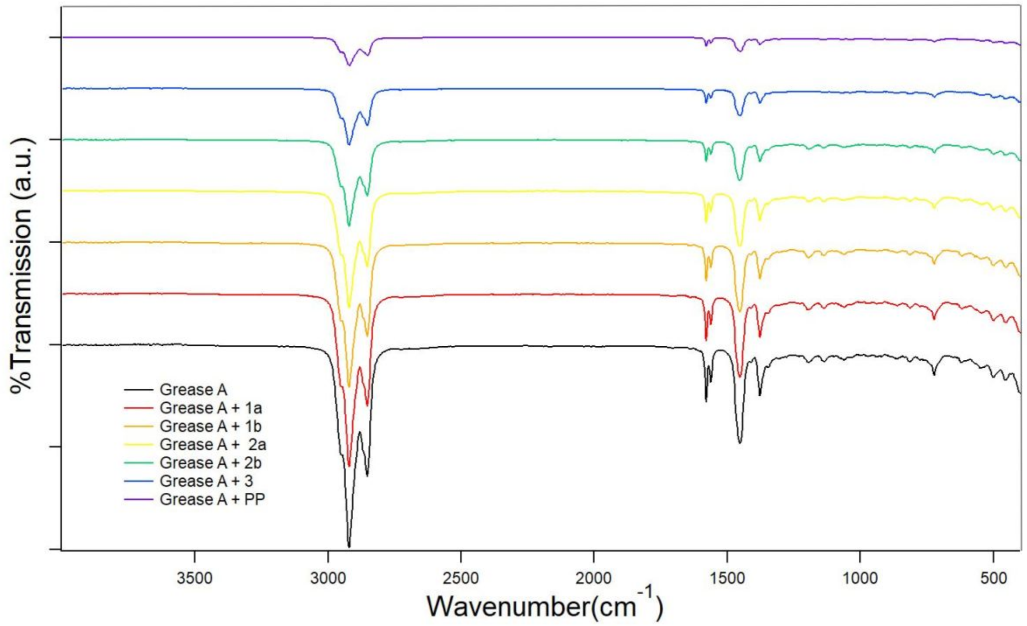

Figure S14. Stacked FT-IR spectra of base and blended grease A with ILs.

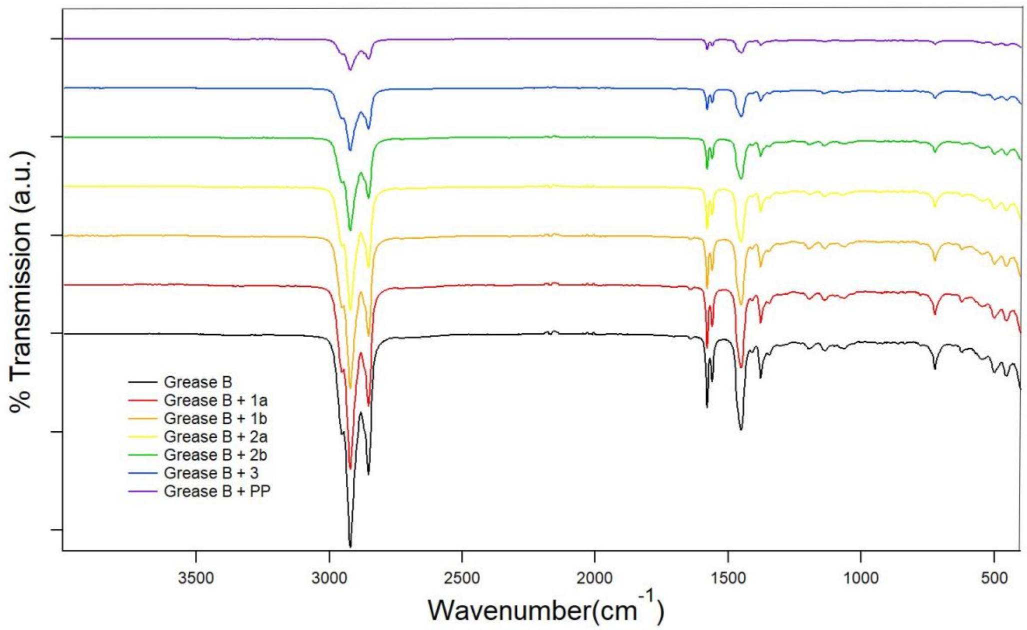

Figure S15. Stacked FT-IR spectra of base and blended grease B with ILs.

TGA of ILs and ILs additized greases.

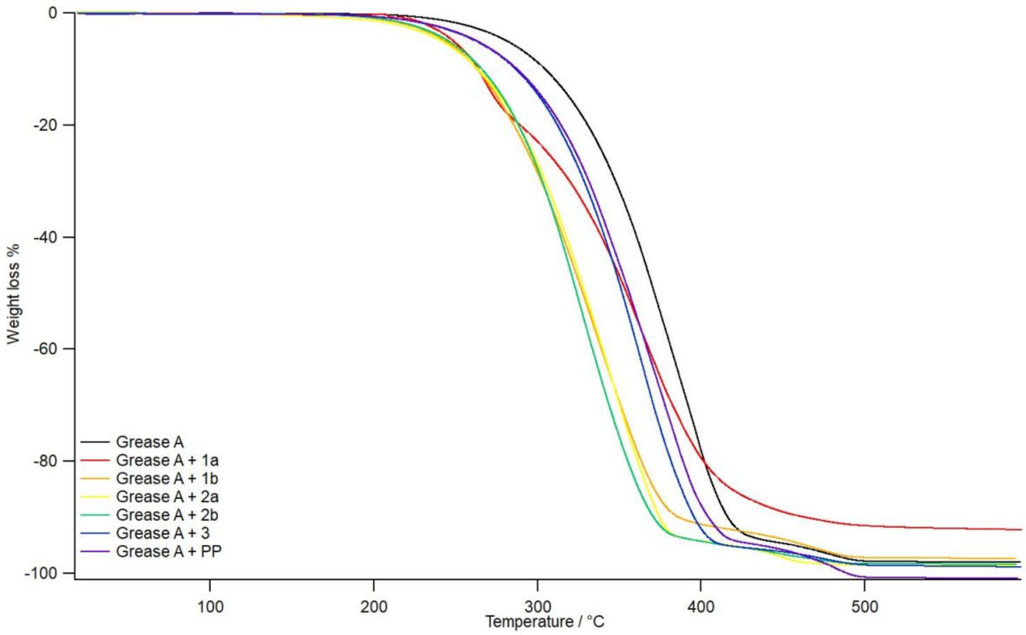

Figure S16. Stacked TGA profiles of base grease B and blended derivatives with ILs

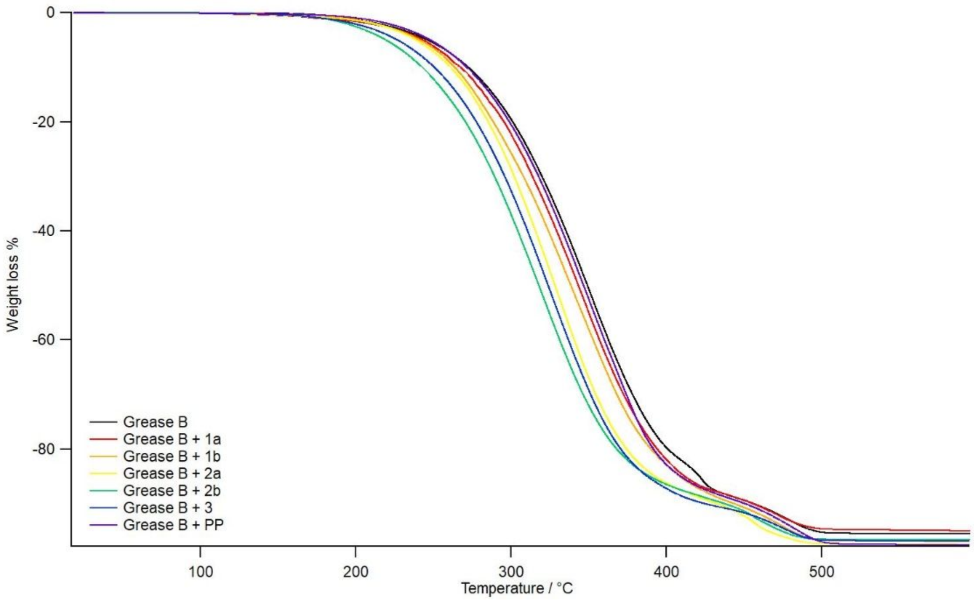

Figure S17. Stacked TGA profiles of base grease B and blended derivatives with ILs.

Schematics and representative raw data from tribological testing.

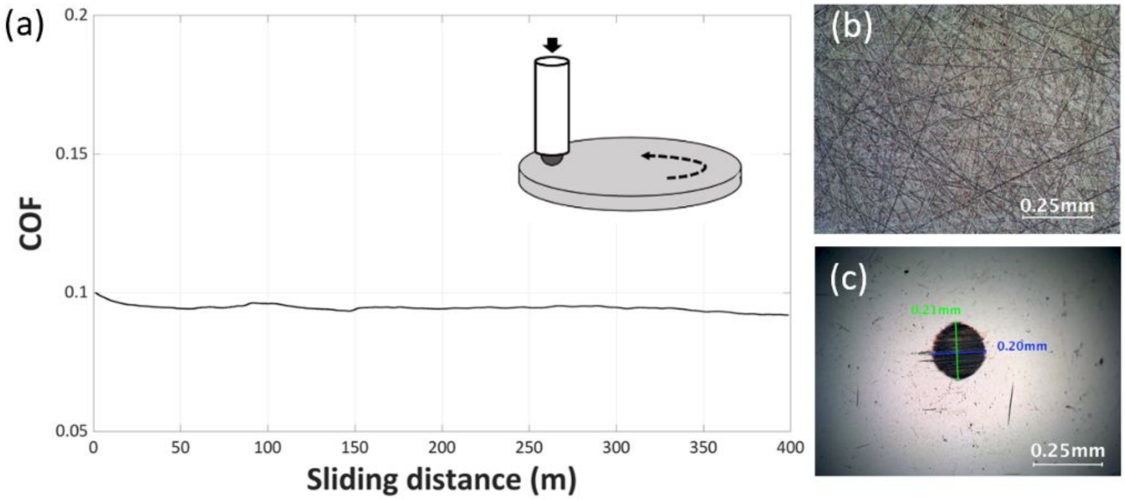

Figure S18: Representative results from a ball-on-disk test. (a) Friction as a function of test time with a schematic of the test set up inset. Optical microscope images of (b) a small section of the wear track on the disk and (c) the circular worn patch on the ball.

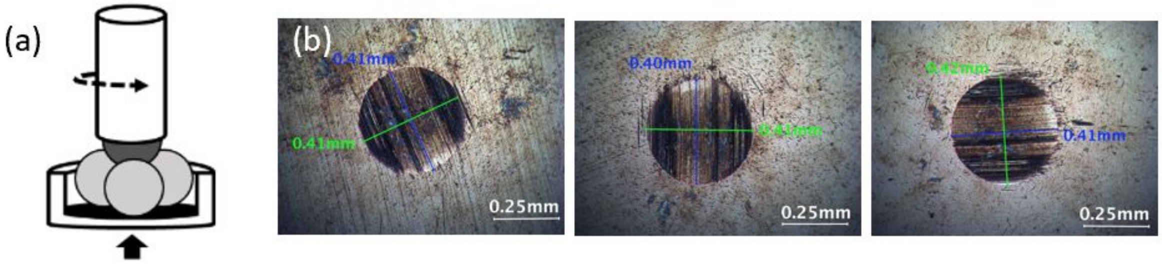

Figure S19: Representative results from a four-ball test. (a) Schematic of the test set up. (b) Optical microscope images of the circular worn patch on the three lower balls that were fixed in place during the test.

Tribological testing friction and wear data.

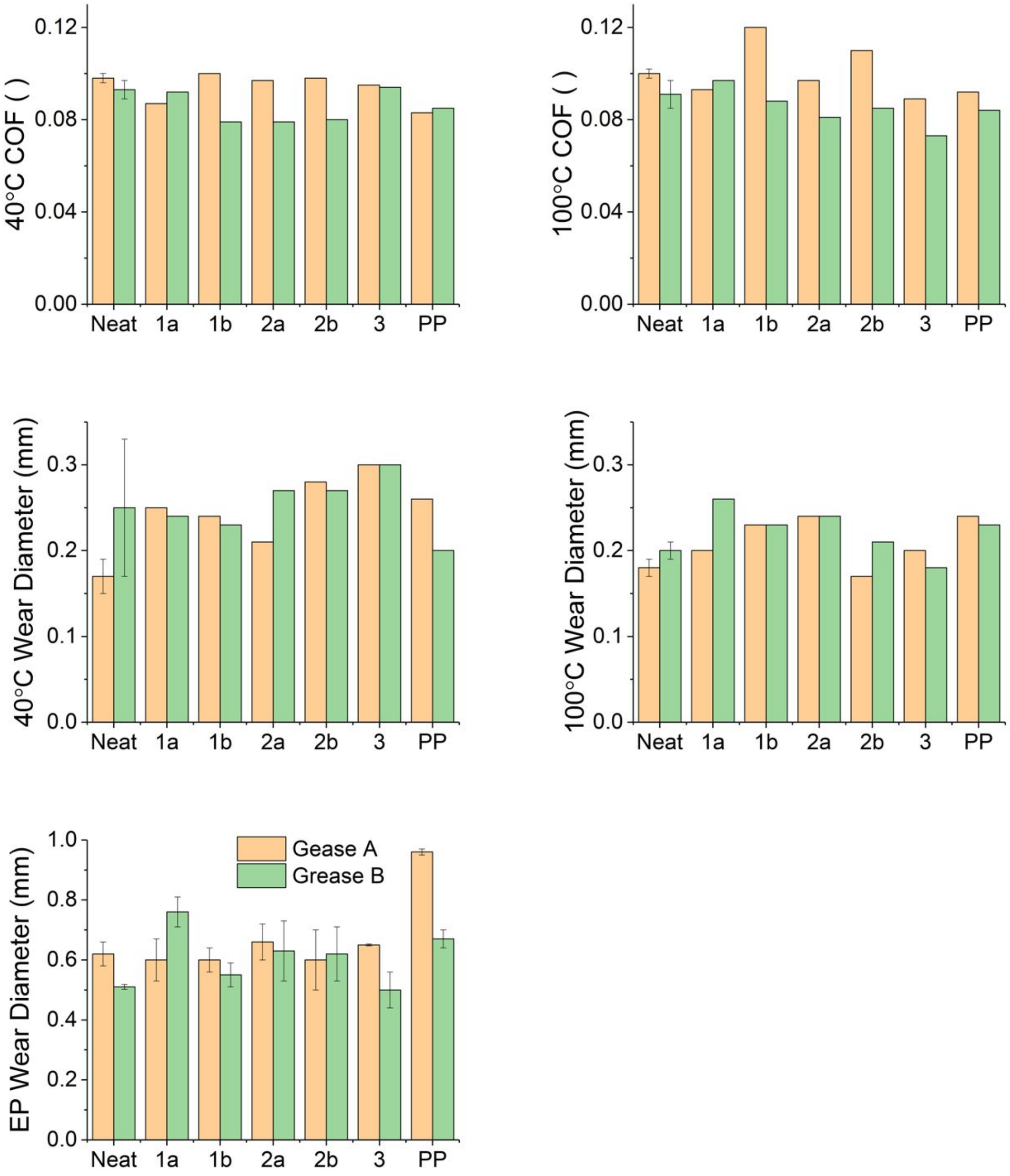

Figure S20: Friction and wear data for the neat and IL-additized greases. When error bars are present, they represent the standard deviation from two separate tests. Data reported with no error bars indicates only one test was run for that grease or condition.

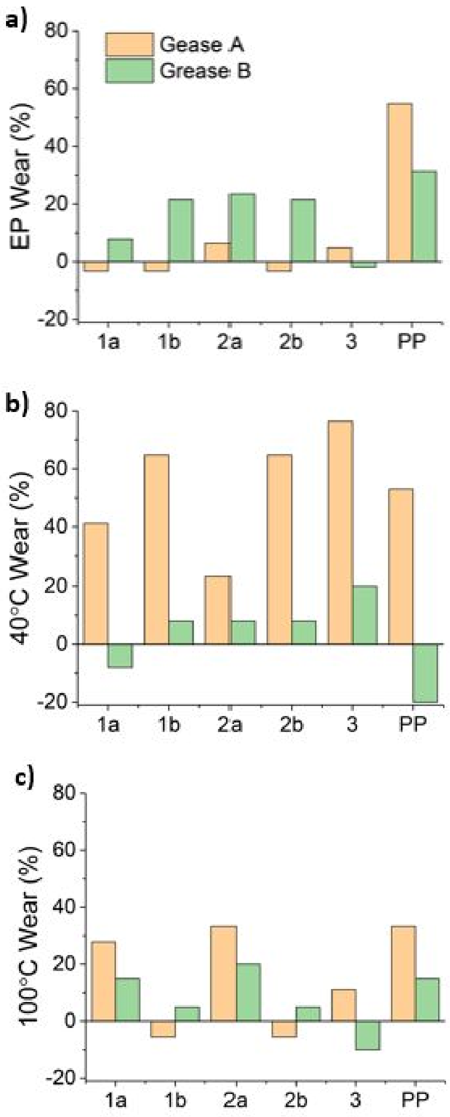

Figure S21. Change in wear diameter from the (a) 4-ball EP test, (b) ball-on-disk test at 40°C, and (c) ball-on-disk test at 100°C resulting from adding the various ILs to grease A and grease B. Negative change indicates performance improvement.

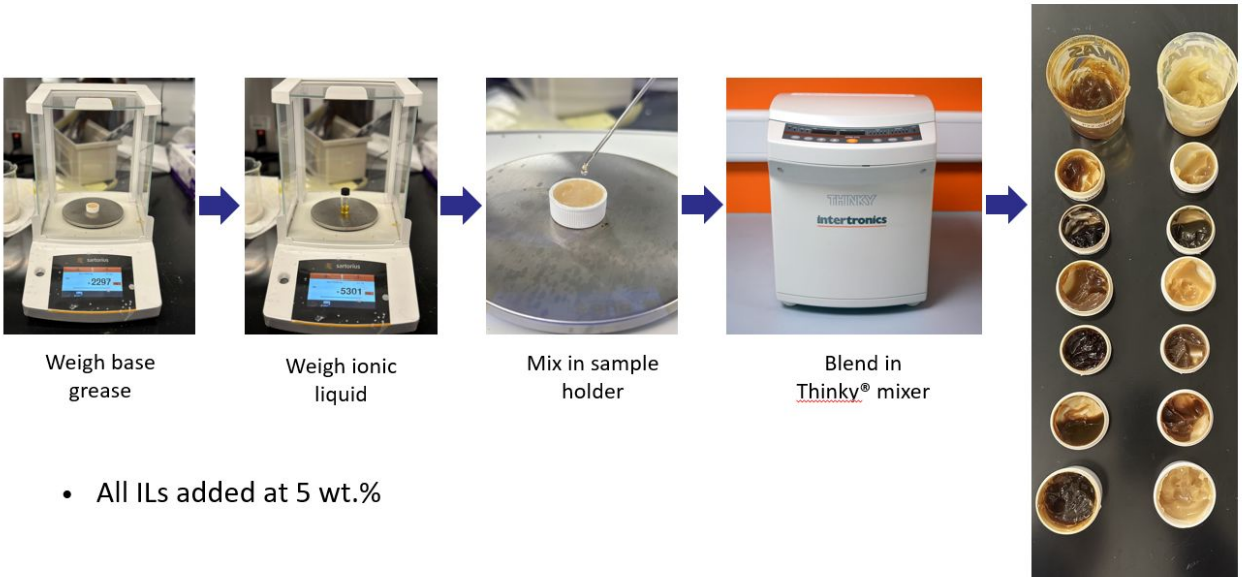

Figure S22: Blending procedure of ILs and base greases.
